# What features of drug treatment programs help, or not, with access? a qualitative study of the perspectives of family members and community-based organization staff in Atlantic Canada

**DOI:** 10.1186/s13011-024-00602-0

**Published:** 2024-03-22

**Authors:** Holly Mathias, Lois A. Jackson, Jane A. Buxton, Anik Dubé, Niki Kiepek, Fiona Martin, Paula Martin

**Affiliations:** 1 School of Public Health, University of Alberta, 3-300 Edmonton Clinic Health Academy 11405 87 AvenueEdmonton, T6G 1C9 Edmonton, Alberta Canada; 2 School of Health and Human Performance, Dalhousie University, Stairs House, 6230 South Street, B3H 4R2 Halifax, NS Canada; 3 School of Population and Public Health, University of British Columbia, 2206 East Mall, V6T 1Z8 Vancouver, BC Canada; 4 Faculty of Health Sciences and Community Services, School of Nursing, Université de Moncton, 18, avenue Antonine-Maillet, E1A 3E9 Moncton, New Brunswick, Canada; 5 School of Occupational Therapy, Dalhousie University, Forrest Building, Room 215, 5869 University Avenue, B3H 4R2 Halifax, NS Canada; 6 Department of Sociology and Social Anthropology, Dalhousie University, Marion McCain Arts and Social Sciences Building, Room 1128, 6135 University Avenue, B3H 4R2 Halifax, NS Canada; 7 Direction 180, 2151 Gottingen Street, B3K 3B5 Halifax, NS Canada

**Keywords:** Family members, Community-based organizations, Drug treatment access, Addiction treatment, OAT, Withdrawal management, Qualitative research

## Abstract

**Background:**

Withdrawal management and opioid agonist treatment (OAT) programs help to reduce some of the harms experienced by people who use substances (PWUS). There is literature on how features of drug treatment programs (e.g., policies and practices) are helpful, or not helpful, to PWUS when seeking access to, or in, treatment. There is, however, relatively little literature based on the perspectives of family members/family of choice of PWUS and community-based organization staff within the context of Atlantic Canada. This paper explored the perspectives of these two groups on what was helpful, or not, about drug treatment programs in Atlantic Canada in terms of supporting access to, and retention in, treatment.

**Methods:**

One-on-one qualitative telephone interviews were conducted in 2020 with the two groups. Interviews focused on government-funded withdrawal management and OAT programs. Data were coded using a qualitative data management program (ATLAS.ti) and analyzed inductively for key themes/subthemes using grounded theory techniques.

**Results:**

Fifteen family members/family of choice and 16 community-based organization staff members participated (*n* = 31). Participants spoke about features of drug treatment programs in various places, and noted features that were perceived as helpful (e.g., quick access), as well as not helpful (e.g., wait times, programs located far from where PWUS live). Some participants provided their perceptions of how PWUS felt when seeking or accessing treatment. A number of participants reported taking various actions to help support access to treatment, including providing transportation to programs. A few participants also provided suggestions for change to help support access and retention such as better alignment of mental health and addiction systems.

**Conclusions:**

Participants highlighted several helpful and not helpful features of drug treatment programs in terms of supporting treatment access and retention. Previous studies with PWUS and in other places have reported similar features, some of which (e.g., wait times) have been reported for many years. Changes are needed to reduce barriers to access and retention including the changes recommended by study participants. It is critical that the voices of key groups, (including PWUS) are heard to ensure treatment programs in all places support access and retention.

## Background

People who use substances (PWUS) are at risk of many health harms including risks of HIV and hepatitis C from injection drug use, and risks of burns and lesions from smoking drugs [1–4]. PWUS may also experience social harms including precarious housing and risk of arrest [5, 6]. In some countries, fatal and non-fatal drug poisonings are increasing, many of which are linked to a toxic drug supply [7, 8]. In Canada, between January 2016 and March 2023, there were 38,514 opioid-related deaths, 37,697 opioid-related hospitalizations, and 16,231 stimulant-related hospitalizations [7]. British Columbia and the Yukon have declared public health emergencies in response to drug-related deaths in their jurisdictions [9, 10].

A comprehensive approach to reducing substance use harms is required. Such an approach must ensure a range of services are readily available across communities (e.g., free syringes and naloxone kits, supervised consumption sites). For individuals who want treatment, access to government-funded drug treatment programs is a critical part of a comprehensive approach. Treatment programs include withdrawal management (also known as detoxification programs and referred to in this paper as detox), and opioid agonist treatment (OAT) which comprises, for example, methadone and buprenorphine or buprenorphine/naloxone (Suboxone®) [11, 12]. In Canada, detox programs are often the ‘first point of contact’ for PWUS who are seeking treatment and these programs help to safely manage medical detoxification of a substance [13, 14]. In some instances, PWUS may be initiated on OAT when in a detox program [15]. Detox programs vary in duration (e.g., a few days to several weeks or months) and content (e.g., varied services such as one-on-one counselling), and are sometimes in-patient programs or outpatient [12, 14]. OAT programs are often viewed as a longer-term form of treatment [16]. OAT programs also vary and can include specialized clinics with an onsite dispensing pharmacy, and physicians and nurse practitioners providing OAT within a private practice [11, 17]. Both detox and OAT programs may offer connections to other psychosocial supports [11, 14].

Research has highlighted various challenges to drug treatment access including stigma related to medication-assisted treatment, and reluctance from physicians to prescribe OAT due to various issues (e.g., inadequate remuneration, provincial treatment regulations, and perceived concerns about diversion of medication) [18–20]. Research also indicates that some treatment programs have features (e.g. policies and practices) that are helpful or act as facilitators to access and retention, as well as features that are not helpful or act as barriers. Our previous research based on the perspectives of PWUS in Atlantic Canada found several features of programs that PWUS experienced as helpful with access and retention including quick and easy program intake, and supportive, non-judgmental program staff [21]. However, some features were identified as not helpful such as wait times, limited program availability, and the stigmatizing attitudes of some program staff [21]. These helpful and not helpful features are not unique to Atlantic Canada. Research conducted in other places, including Colombia, Ukraine, and the United States, have identified helpful features, such as easy access to OAT and supportive program staff, and not helpful ones, including wait times, complicated intake processes and limited availability of programs [22–24]. Understanding the perspectives and experiences of PWUS is critical to identifying what features of drug treatment programs are helpful or not helpful but it is also important to understand the perspectives of key groups (e.g., family members of PWUS, community-based organizations), given their knowledge and experience supporting or working with PWUS who are seeking or in treatment. As Adam and Kitt-Lewis (2020) note, “The opioid epidemic is a complex problem. In order to provide a comprehensive intervention plan, understanding all stakeholders’ perspectives is essential” (p.475) [25].

The key purpose of our qualitative research was to understand, based on the perspective of two groups (family members/family of choice of PWUS and community-based organization (CBO) staff who work with PWUS), features of drug treatment programs that are helpful or not helpful for PWUS when trying to access or accessing government-funded treatment programs in Atlantic Canada. There is some existing research on the support which family members/family of choice and CBO staff sometimes provide to PWUS when they are seeking or in treatment [25–27], but relatively limited research on these groups’ perspectives of features of drug treatment programs that are helpful or not. The limited literature that does exist on family perspectives points to quick access as being helpful [28], and not helpful features include limited availability of services, cost of services, and wait times [27–29]. Most of this literature, however, centres on youth treatment programs [27, 29, 30]. CBO staff perspectives on drug treatment programs are also relatively underexplored [31], and there is specifically a knowledge gap based on treatment programs in Atlantic Canada. Our research aimed to help fill some of these current gaps in the literature.

## Methods

### Study setting

Atlantic Canada is a region in eastern Canada comprised of four provinces (New Brunswick, Newfoundland and Labrador, Nova Scotia, and Prince Edward Island) with a total population of approximately 2.6 million people across a geographical area of 504,320km^2^ [32, 33]. The Atlantic region has the largest rural-dwelling population in Canada with nearly 50% of the population living rurally [34, 35]. It is estimated that in 2016, between 10,660 and 13,370 people injected drugs in Atlantic Canada [36]. From January 2016 to March 2023, 889 drug-related deaths were reported in the region [7]. Although health services can vary by province at the time of our study all four provinces had government-funded detox and OAT programs [11, 14]. Exactly how many individuals have accessed or are accessing drug treatment programs in the region is unknown.

### Study design

The data reported in this paper are drawn from Phase Two of a three-phase study developed in partnership with community knowledge users, as well as researchers from across Atlantic Canada and beyond (See more about team composition and study design in Jackson et al., 2022 [21]. The focus of each phase of the study was on detox and OAT treatment programs in Atlantic Canada. Phase One explored the perspectives of PWUS [21], and Phase Two, which is the focus of this paper, explored the perspectives of family members/family of choice of PWUS and CBO staff working with PWUS. The relevant institutional research ethics boards approved the study.

### Participants

#### Eligibility

Individuals were eligible to participate as a family member/family of choice if they reported that they were a family member/family of choice of someone using substances, were 19 years of age or older, and were able to speak to the experiences of their family member when they were trying to access or accessing government-funded drug treatment programs in Atlantic Canada (i.e., detox and/or OAT) within approximately the previous two years. CBO staff were eligible to participate if they were 19 years of age or older, a current paid employee (full-time or part-time) for approximately one year at a community-based organization providing harm reduction services in Atlantic Canada, and able to speak to their clients’ experiences when accessing or when in government-funded drug treatment programs within approximately the previous two years. CBO staff participants were from the organizations that had executive directors involved as knowledge users on the research team [21]. Generally, the CBOs provided a range of harm reduction services, including access to syringes and other safer use equipment, education, outreach, service navigation and referrals, and in one instance, an on-site OAT dispensary. CBOs were located across the Atlantic region with at least one participant from each province. It is important to note that although participants were asked to speak about PWUS (i.e., individuals who inject drugs and/or smoke crack cocaine use) who had tried to access or accessed treatment within the two years prior to the interview, some participants spoke about a more extended period of time. Knowledge users and collaborators on the research team at the time of the study were not eligible to participate.

### Recruitment

Purposeful sampling was used to recruit family members/family of choice and CBO staff. Recruitment occurred through seven community-based harm reduction organizations across the four Atlantic provinces. Knowledge users (executive directors of the organizations) recruited family members/family of choice by directly approaching individuals known to them (via phone, email or in person). Knowledge users also distributed study recruitment posters to family members/family of choice, and to PWUS to share with their family members, and posted recruitment information through their organization’s social media pages (e.g., Facebook, Twitter). CBO staff were recruited through the knowledge users who shared the recruitment poster internally with staff. CBO staff were also recruited through snowball techniques (i.e., staff telling each other about the study). The knowledge users helping with recruitment were encouraged to recruit individuals from various backgrounds (e.g., different gender identities and age groups). Interviews were scheduled as individuals who met the eligibility criteria volunteered to participate. Interviews took place between March and September 2020.

Individuals interested in participating emailed or telephoned (toll-free number) the study research co-ordinator to express interest. The research coordinator (HM) reviewed a screening document with the participant to ensure eligibility.

### Data collection

One-on-one telephone interviews were used to collect data. Before each interview, the interviewer (HM) reviewed the informed consent form, answered questions, and obtained verbal consent. Participants were informed that their access to services and/or employment would not be impacted by their participation or what they shared during the interview. All participants were provided a $20 CAD e-gift card as an honorarium. Semi-structured interview guides were developed for family members as well as for CBO staff. The guides were developed in collaboration with the research team. One research team member with experience of having a family member who uses substances piloted the family member interview guide. Two knowledge users on the team working in a community-based organization piloted the community-based staff interview guide. Some changes to the wording of questions and the probes provided were made based on their feedback.

The interview guide included questions about drug treatment program (e.g., policies and practices) that were helpful or not helpful in terms of access and retention. For example, family members/family of choice were asked: What do you perceive your *family member* found helpful when trying to access OAT? Examples of probes were: ‘Was there support for transportation or childcare? Were the program staff supportive?’ The interview guide also included questions about how participants perceived the PWUS felt when seeking treatment and how drug treatment programs might be improved. In addition, participants were asked if they perceived any changes to PWUS’ drug use or safer drug use practices when trying to get into treatment/in treatment, but these data are *not* reported in this paper. A few participants also spoke about some COVID-19-related issues that are not part of this paper. At the end of the interview, participants were asked a few sociodemographic questions.

Interviews were audiotaped with the permission of the participant. If the participant did not want to be audiotaped, the interviewer took notes by hand. Four participants opted for handwritten notes. Each interview lasted between 20 and 60 min. Audio-recorded interviews were transcribed by a research assistant and checked for accuracy by the research coordinator, and handwritten notes of interviews were typed verbatim into a word-processing program.

### Data analysis

The transcripts and written notes were entered into a qualitative software program (ATLAS.ti) and organized by family member/family of choice or CBO staff, and province, with each of the four provinces given an identification letter (e.g., Site A). The research coordinator (HM) inductively coded a couple of transcripts using the analytic techniques of comparing and contrasting key concepts within and across the interview transcripts [37, 38]. The preliminary codes (e.g., time) were discussed with a team member (LJ) and the larger research team. The finalized coding structure was utilized to code all transcripts, and a summary of the coded data and developing themes were discussed with the larger team. HM and LJ further developed key themes and sub-themes through a review of the coded data (often returning to the full interviews to understand the broader context of the coded data) and discussed the final themes with the paper authors.

Lincoln and Guba (1985) suggest there are various strategies that can help ensure trustworthiness of findings [39]. For this study, members of the research team were involved in discussing research findings, and a number of these individuals had several years of experience working with PWUS. The authors of the paper also further discussed the data and findings. To support possible transferability of findings, we have provided a detailed description of our methods and data analysis, as well as the context of the study.

## Results

Fifteen family members/family of choice, and 16 CBO staff, were interviewed (*n* = 31). Most of the 15 family members/members of choice identified as women (*n* = 12, 80%) and reported living in a city (*n* = 12, 80%), with all identifying as white/Caucasian (*n* = 15, 100%) (see Table 1). Family members were not asked to specifically identify their relationship to the person using substances but during the interviews a number indicated that they were a parent/stepparent, sibling, partner, or boyfriend/girlfriend.

**Table 1:** Sociodemographic information as reported by interviewed family members and family of choice of people who use substances in Atlantic Canada (*n* = 15)

Sociodemographic Information	n	%
Gender		
Woman	12	80.0
Man	3	20.0
Ethnicity		
White/Caucasian	15	100.0
Age Range		
19–29	4	26.7
30–39	2	13.3
40–49	1	6.7
50–59	4	26.7
60–69	4	26.7
Live in a City		
Yes	12	80.0
No	3	20.0

Of the 16 CBO staff, the majority identified as women (*n* = 15, 93.8%) and most lived in a city (*n* = 14, 87.5%) (Table 2). Over half of the CBO staff participants had worked at their organization for 2 to 5 years (*n* = 9, 56.3%).

**Table 2:** Sociodemographic information as reported by interviewed community-based organization staff in Atlantic Canada (*n* = 16)

Sociodemographic Information	n	%
Gender		
Woman	15	93.8
Man	1	6.2
Live in a City		
Yes	14	87.5
No	2	12.5
Time spent working at organization (in years)		
1 year or less	2	12.5
2–5 years	9	56.3
6–9 years	2	12.5
10 years or more	3	18.7

Four key themes were generated from the interview data. Theme 1 highlights features of some drug treatment programs (e.g., policies) that participants perceived as helpful when individuals were seeking, or in, treatment, and theme 2 outlines features that were perceived as **not** helpful. It is important to note that programs vary across the region, and that some programs may have both helpful and not helpful features. Themes 1 and 2 also include a discussion of what participants perceived as PWUS’ emotions when seeking, or in, treatment. Theme 3 draws attention to a few key actions participants reported that they took to help with access to treatment. Theme 4 outlines some participants’ views of what needs to change to help support individuals seeking treatment.

Quotes from interviews are presented by site (e.g., Site A), family member/family of choice (FA) or CBO staff member (CB), and corresponding participant number.

### Theme 1: Features of drug treatment programs that help when seeking or in treatment

#### Seeking treatment

A few drug treatment program policies and practices were identified as helpful to individuals when seeking treatment including readily accessible programs. A family member reported that there was quick access to OAT for the individual in their family seeking treatment, and a couple of CBO staff also highlighted quick access including “self-referrals” (Site D, CB#5). Allowing an individual to restart OAT treatment with “no problem” after they had stopped for a period of time was also identified as a helpful practice (Site A, CB#1), as was “physician support” (Site B, CB#1).

When asked about what they believed their family member/family of choice or clients were feeling when seeking treatment, a couple of participants spoke about a number of different emotions, both positive and negative. A CBO staff member commented that some individuals not only felt “excited” but “terrified” at the same time, and that there were, “…a myriad of emotions because one minute you want to go, the next minute you’re thinking you don’t… (Site A, CB#3). Referring to detox programs, another CBO staff member indicated that some individuals were fearful because, “They are not sure how long the detox is going to last or even if they are going to lose their job, if they have a job. Or if they’re going to lose their apartment…” (Site C, CB#3). A couple of CBO staff members also commented that some pregnant women or parents of small children feared the involvement of children aid services.

#### In treatment

A number of participants spoke to the importance of programming when in treatment including wrap-around supports (e.g., access to a social worker), one-on-one counseling, and drop-in counseling. A CBO staff member indicated that counselors with “life experience in the world of addiction” were helpful (Site A, CB#5). Having a “safe place [in detox] where they can talk about [their] feelings” was also noted as helpful by a CBO staff member (Site C, CB#2). According to yet another CBO staff member, many individuals in detox need support with trauma, and “somebody to listen to them” (Site C, CB#3). Providing individuals with a space in a detox program to “reset” and perhaps return another time to try treatment again was highlighted by a CBO staff member who stated that, “People get to go somewhere and be well fed and have a place to sleep and feel safe and have support. Even if it’s just a week…It kind of resets you to come back and tackle it again, right?” (Site D, CB#2).

Connecting individuals to mental health supports was identified as helpful for OAT retention by a CBO staff member, and according to a family member, group sessions helped with methadone retention for the individual in their family. A CBO staff member stated that “adjusting” or changing the clinic or pharmacy that the person uses can help with retention if the person is not happy at a particular clinic or pharmacy (Site C, CB#1), and a family member commented that having a pharmacy close to where the individual lives was helpful. A couple of CBO staff spoke about helpful transportation services (e.g., a publicly-funded medical transportation program). Carries or take-home doses of OAT were also spoken of as helpful as individuals did not have to travel daily for OAT treatment, suggesting that carries supported retention for some individuals.

A number of family members and CBO staff referred to treatment staff that were supportive, non-judgemental or “good” (Site A, FA#5). It was not always clear if there were links between supportive staff attitudes and retention, but the importance of positive staff attitudes was highlighted. A few participants provided specific examples of staff practices that they believed were helpful including a physician with a “compassionate approach” “where clients felt safe” to talk to the physician (Site D, CB#2), OAT physicians on call on weekends, and “daily contact” with a nurse or case manager for individuals on OAT without family or many friends (Site A, CB#1). A family member spoke about a pharmacist who was helpful as they remained overnight at a pharmacy during a snowstorm to ensure methadone could be distributed the next day. A CBO staff member maintained that peer support between individuals in treatment was also helpful, and they stated that:

…peer support. That’s a huge thing that we see in our space. People who know each other and know that each other are on methadone are often really supportive of one another…We’ve seen really beautiful moments of peer support where, for example, if a woman is accessing her pharmacy and her abusive ex-partner also has to access that pharmacy…other women they know help them get in and out of the pharmacy to remain a bit safer (Site D, CB#3).

Some participants spoke about their perceptions of the various emotions individuals had when they were in treatment, and one participant indicated that their family member missed people when in detox. A CBO staff member noted that “relief” is an emotion some individuals experienced when in a methadone program (Site D, CB#3), and another CBO member stated that when in treatment some individuals reported that they felt “supported” and “hopeful” and some felt “stuck” because “they didn’t realize the expectations and that kind of ties them down” (Site D, CB#1). According to one CBO staff member, some individuals felt “empowered” when they were in an OAT program as they gained access to their children, maintained employment, or went back to school (Site A, CB#1).

### Theme 2: Features of drug treatment programs that do not help when seeking, or in, treatment

#### Seeking treatment

A couple of family members indicated that there were challenges trying to obtain information about treatment. Having to phone (sometimes daily) to determine if there was a bed in detox was also identified as not helpful by several family members as well as CBO staff. Some PWUS seeking treatment do not have a phone, and as a CBO staff member commented, phoning “is a huge barrier because some clients don’t have a phone to make the initial phone call…” (Site D, CB#2). Another CBO staff member stated that even if an individual uses the CBO agency phone for the initial call there is not always someone at the agency to receive the call back message from the treatment site.

A couple of CBO staff members indicated that the process for accessing treatment was often confusing. Wait times for some detox and OAT programs were also highlighted by a number of CBO staff as well as family members as not helpful, and one family member commented that, “…The wait times are crazy…For someone who’s actually reaching out for help to find out they’re going to have a bed [in detox] next Thursday is just not going to work” (Site A, FA#3). A number of participants also spoke about the emotions that some PWUS have when waiting for treatment including “frustration” (e.g., Site A, FA#3), “desperation” (Site B, CB#1), and a “rollercoaster” of emotions including anger (Site A, FA#3). A CBO staff member noted that waiting for a detox program can be “discouraging” (Site C, CB#2). Yet another CBO staff member maintained that wait times for treatment can feel like an “eternity”, and this participant stated that, “…when they [individuals seeking treatment] come forward and want support, if we don’t help them like, in that moment when they’re ready, we lose people. And that could mean we lose people to death or we lose people to incarceration or we lose people to relapse” (Site D, CB#2).

A few CBO staff members indicated that in their community there were a limited number of detox programs or detox beds which created access challenges, and one staff member stated that in their community they have “done away” with admitting individuals who have opioid use disorder to detox “and starting them on a medication then weaning them off given that when they are released they are at higher risk of overdose or death” (Site A, CB#1). A few participants pointed to the limited number of physicians available within their communities who prescribed OAT, and one CBO staff member reported that “some days” individuals in their community were not able to access OAT because there were no physicians available (Site A, CB#5). Another CBO staff member commented that, “…not having any doctors willing to take you on [for OAT] can sometimes set people back an awful lot” (Site D, CB#1).

#### In treatment

Family members and CBO staff noted that the geographical location of a drug treatment program sometimes meant travel, including travel in poor weather, which could be challenging. A CBO staff member stated that the detox program in their community was not that “far if you have a vehicle” but if you must walk it is quite a distance (Site C, CB#2), and a family member commented that detox was “on the other side of town” and taking buses was time consuming (Site C, FA#3). For some individuals travel was also reportedly financially costly, and a CBO staff member noted that some individuals on OAT spend a lot of time traveling to a pharmacy. This participant commented as follows:

There’s only 3 pharmacies here [in their community] that dispense…2 of them aren’t open on the weekend. So if you’re banned from one [of] them [pharmacy] for example, which is the one open on weekends, but you’re not yet eligible to go to the other pharmacies because you can’t get carries and they’re not open on the weekend….we now have people who have to drive as far as [town] to get their prescription. That’s an hour to and from so two hours total every single day to get that. And you need to do that on your own dollar….You can imagine that that presents a huge challenge for people trying to stay on the program (Site D, CB#5).

One family member stated that challenges accessing a bus pass led their family member to discontinue OAT, and according to a CBO staff member a client “walked away” from treatment because of the cost of travel (Site A, CB#4).

A few family members and CBO staff spoke about the challenges of daily OAT treatment, as well as wait times for appointments. A CBO staff member stated that, “People that work and then they have to go literally, if they don’t [have] carries, they have [to] go daily [to access treatment]…do we make other people do that for their prescriptions?” (Site C, CB#2). Another CBO staff member indicated that the hours of operation of clinics/pharmacies sometimes added to the challenges of daily access (Site D, CB#5), and a family member noted that the individual in their family on OAT would “almost go into a panic” because of the limited hours of the pharmacy and fears related to possibly not accessing treatment (Site C, FA#1). A couple of other family members noted that at times a daily dose of OAT was missed because of the limited hours of operation of a clinic/pharmacy or because travel was not possible. A CBO staff member stated that stable housing was a condition for accessing carries, and they noted that, “…the barrier to getting carries is definitely stable housing. If you’re couch surfing or you live in a shelter…It’s like okay, we would really like to give you some carries…But you live in an unstable housing situation” (Site A, CB#5). The financial costs of accessing treatment were also mentioned by a couple of participants including the costs of methadone for some (e.g., if not on income assistance). A CBO staff member stated that PWUS who are parents sometimes had to pay for childcare to access treatment.

No cigarette smoking policies in detox were highlighted as not helpful, and one CBO staff member stated that, “Some people smoke [cigarettes]. They want to go out for a smoke and they’ll leave [detox] because they can’t have one, which sounds kind of small, but when you’re in a situation like that, the last thing you want to be told is that you can’t have a cigarette” (Site A, CB#6). A couple of CBO staff members indicated that some PWUS do not enter detox if they learn that there is a no smoking policy.

Limited time in detox was also spoken of as not helpful by a couple of participants, and in some places, the limited time given to mental health issues (e.g., limited counseling) was raised as not helpful. Speaking about a detox program, a CBO staff member commented that, “…they see a psychologist or a counsellor maybe once in a week, and then they end up being sent home with no follow-up…” (Site A, CB#6). Another CBO staff member indicated that OAT programs in their community had a lack of “wrap around supports and talk therapies that go with that” (Site B, CB#1).

Negative, unsupportive, or stigmatizing staff attitudes were commented on by a few family members as well as CBO staff members. Speaking about a methadone program, a CBO staff member stated that they had heard that there was “not a welcoming kind of feeling from the office staff…And even the doctor” (Site D, CB#4).

According to a CBO staff member, PWUS have various feelings such as frustration, anger or even shame when they are not able to stay in treatment (Site A, CB#1). A family member stated that when their family member was unable to stay in treatment they came home “deflated” and felt “hopeless” (Site A, FA#3). Yet another family member commented that the person in treatment was “mad” because they wanted to stay in treatment longer (Site A, FA #6).

### Theme 3: Taking action to help with access to treatment

A few family members and CBO staff indicated that they sometimes engaged in various actions to help with access to treatment or help address a feature of a drug treatment program (e.g., policy or practice) that was not helpful. A couple of family members reported, for example, that they searched for information about drug treatment programs when such information was not readily available, and a CBO staff member spoke about PWUS sometimes having a “discussion” about treatment with CBO staff because information is not “out there enough” (Site A, CB#3).

A family member noted that they provided the individual in their family seeking treatment with access to a telephone so that they could contact a drug treatment program, and a few CBO staff members reported that PWUS sometimes used the telephone at their agency to contact a treatment program. A few family members and CBO staff also helped with transportation to treatment (e.g., a detox program, the pharmacy). A couple of CBO staff members indicated, for example, that their organization sometimes provided individuals with bus tickets or taxi chits/coupons to get to a treatment site, or someone from the agency might drive a client to detox or to appointments. A CBO staff member commented that, “If one of our clients told us they wanted to go to detox but they couldn’t get there, one of us would drive them or we’d put them in a cab or whatever the case is. Make sure they get there and to their appointments” (Site A, CB#4). Another CBO staff member stated that, “…I’ll drive you there [to detox] and leave you with a bus token to get home…[but] the system should be set up to accommodate people’s needs. Not us trying to fit in to the system…” (Site C, CB#1). A couple of CBO staff also noted that they sometimes helped individuals complete forms such as forms to obtain subsidized transportation that helped with travel to access treatment.

### Theme 4: What needs to change

A few family members and CBO staff pointed either implicitly or explicitly to changes that were needed to reduce if not eliminate features of drug treatment programs within their community that were not helpful for individuals seeking or in treatment. A couple of participants suggested, for example, that treatment should be available as soon as the person wants treatment or the day an individual wants treatment, and that limited time in detox should be changed to allow longer stays. Addressing travel costs and having drug treatment programs within close physical proximity to where individuals live were two changes implicitly suggested given the travel challenges outlined by a number of participants. One family member maintained that a bus pass should be provided the day an individual begins OAT in places where there are buses. A CBO staff member noted that within their community some individuals have costs associated with carries but having carries “free” and “more widespread” would help reduce travel time to a pharmacy/clinic thus helping with individuals’ employability as well as their ability to be involved with various community programs “which otherwise are not top of their priority because their priority is getting back and forth to the clinic” (Site D, CB#3). A family member emphasized the importance of supportive and non-judgemental treatment staff and maintained that there “needs to be re-education for everyone who works in addiction, and people need to stop looking at people who use as criminals” (Site B, FA#1).

#### Filling treatment service gaps

A couple of family members and CBO staff maintained that there were “gaps” in treatment services that needed to be filled. One CBO staff member pointed, for example, to the gap in services or long wait times between leaving detox and entering a “recovery” program (Site B, CB#1), and another noted that in their community there was no option for OAT. A CBO staff member also maintained that for a number of individuals on OAT with a “private doctor”, “they’re not getting any kind of supplementary addiction counseling. So that’s a big gap” (Site D, CB#4).

#### Better linkages across services and systems

A couple of participants spoke about a need to create changes that would ensure better linkages across services including a need to have better linkages between mental health and addiction services. Referring to a detox program, a family member maintained that there was a need for “…more of the mental health social workers for everybody to talk to afterwards [after detox]. Because they come out of there and it’s just like throwing them under the bus….they need more mental health [supports] (Site A, FA#6). Better alignment with pharmacy services was also implicitly suggested given the challenges for people on OAT when pharmacies were closed or had limited hours. A couple of CBO staff members also indicated that there was a need for childcare supports for parents seeking treatment or in treatment, and supportive housing for some individuals after they leave treatment. These changes highlight a need for better linkages with different systems including community services and the housing sector.

## Discussion

Family members and CBO staff identified several features of different drug treatment programs in Atlantic Canada that they perceived as helpful, and not helpful, for individuals when seeking access to or in treatment. PWUS in our previous research from Phase One of the study identified a number of similar features that were helpful (e.g., quick access, supportive staff) and not helpful (e.g., needing a phone to obtain access, wait times, limited program availability) [21]. Many of the policies and practices highlighted by family members/family of choice and CBO staff and presented in this current paper on Phase Two data, have also been identified in the literature for many years [27, 28, 31, 40, 41].

A few family members and CBO staff in our research were reportedly active in helping to address a few policies and practices that were not helpful such as providing access to a phone in instances where a phone was necessary to access a treatment space. These findings add to the body of literature on family and CBO support for PWUS when accessing treatment, including literature which indicates that these groups sometimes help with providing transportation to treatment [42–45]. According to the literature, providing support may have personal impacts on those providing the support, and research has found, for example, that some family members report physical and mental health issues (e.g. chronic illness, anxiety) linked to supporting their family members who are accessing addiction treatment [27, 46].

Participants in our research pointed to the need for changes to some drug treatment programs including the need for immediate access to treatment without waiting and better program availability in places where current treatment requires extensive travel. Such changes might reduce the need for family members and CBO staff to be involved in supporting PWUS when accessing treatment. The need for these types of changes has been discussed in the literature for many years [47, 48], and a key reason why such changes have not happened may be, in part, because of the historical problem of underfunding for drug use services [49–51]. Increased funding for services for PWUS could potentially reduce, if not eliminate, treatment wait times where they currently exist, and improve program availability across communities thus reducing travel costs and the time required to access treatment experienced by some PWUS. Increased funding might also help to ensure ongoing staff training to promote supportive and non-judgmental attitudes among those providing treatment services. All four of the Atlantic provinces have referred to increased funding for mental health and addictions in their 2023-24 budgets. This is a positive sign, but it is important to note that only New Brunswick explicitly references funding treatment programs, and it is not clear if the funded treatment programs will be for mental health, addictions, or both [52–55].

In some instances, there are economic costs associated with OAT, and specifically treatment medication [56]. British Columbia recently announced that the provincial universal healthcare plan will cover the cost of OAT medication for everyone regardless of their situation (e.g., on social assistance or not) [57]. This change in policy provides “immediate, barrier-free access to OAT medication” [57], and if implemented across Atlantic Canada (as well as elsewhere) would help with treatment access and retention.

Changes in policies and practices are also needed to facilitate the need for better service and system linkages identified by participants in our research. Specifically, there is a need for better linkages between mental health and addiction services sometimes referred to as ‘integration’ in the literature [58], and better alignment with other systems (e.g., housing sector). The need for better linkages across systems and, thus, a reduction in ‘silos’ has been highlighted by many other researchers for some time [59–61]. Family members and CBO staff in our study identified transportation needs and childcare responsibilities as impacting treatment access and retention, thus pointing to the importance of implementing free, accessible public transportation, and providing accessible childcare supports. Access to supportive housing following treatment was also identified as critical and points to the importance of better linkages with the housing sector.

Changes to existing drug policies may also help to reduce some of the barriers to treatment identified not only by some participants in our study but also the wider literature. Decriminalization may lessen the stigma experienced by PWUS, including stigma from healthcare providers, reducing one potential barrier to accessing treatment [62–64]. Decriminalizing drugs may also help governments shift funds away from drug law enforcement to enhance drug treatment program access [64]. The province of British Columbia has decriminalized the possession of a small amount of drugs for personal use [65] and some other cities in Canada, such as Toronto, are considering following suit [66] pointing to a mounting interest in creating changes to current drug policies.

Families and CBO staff may play an essential role in advocating for changes needed to ensure all drug treatment programs support access and retention. Indeed, the voices of these groups, and those of PWUS, are critical to creating urgently needed program and policy changes [67, 68].

### Study limitations

Our findings contribute to the existing knowledge about helpful and not helpful features of drug treatment programs but there are a number of study limitations specifically related to our recruitment strategy. Family members/family of choice were recruited through community-based organizations, and individuals who met the eligibility criteria were interviewed. Most who volunteered identified as white/Caucasian and as women. It is not known how diverse the population of family members/family of choice might be but recruitment through other venues (e.g., treatment programs) might have resulted in a more gender and racially-diverse sample of individuals with different perspectives.

Most of the participants in our study (family members/ family of choice, and CBO staff members) reported that they lived in a city. Having a targeted recruitment strategy to specifically access individuals living in rural and remote places might have also add to the diversity of the sample and thus different or additional perspectives to those highlighted in our study.

## Conclusions

Family members and CBO staff identified several features of drug treatment programs in different places in Atlantic Canada that they perceived as helpful or not helpful for PWUS when accessing treatment. Some family members and CBO staff indicated that they tried to help address a few of the features that were not helpful, including providing access to a phone for treatment access and helping with transportation to treatment. A few participants argued that changes were needed to drug treatment programs such as having immediate access to treatment, and that systems linked to drug treatment programs needed to work in unison, including the mental health system. Family members and CBO staff may be key advocates not only for such needed changes but also for the funding that is likely required to support the changes.

## Data Availability

Study participants did not agree for their data to be shared publicly as part of the consenting process, so data are not publicly available.

## Abbreviations

CBO: Community-based organization
OAT: Opioid agonist treatment
PWUS: People who use substances
